# Peripheral Oxidation Markers in Down Syndrome Patients: The Better and the Worse

**DOI:** 10.1155/2021/5581139

**Published:** 2021-06-28

**Authors:** Dominik Szwajgier, Ewa Baranowska-Wójcik, Joanna Grzelczyk, Wioletta Żukiewicz-Sobczak

**Affiliations:** ^1^Department of Biotechnology, Microbiology and Human Nutrition, University of Life Sciences in Lublin, Skromna 8, 20-704 Lublin, Poland; ^2^Institute of Food Technology and Analysis, Faculty of Biotechnology and Food Sciences, Łódź University of Technology, 90-924 Łódź, Poland; ^3^Department of Food and Nutrition, Calisia University, Nowy Świat 4, 62-800 Kalisz, Poland

## Abstract

Oxidative stress plays an important role in Down syndrome (DS) pathology since the gene dose effect leads to abnormal levels of certain enzymes and metabolites. In this review, we focused on relatively easy-to-obtain, peripheral markers of oxidative stress and inflammation, in order to compare the levels of these markers in DS patients and chromosomally healthy persons. Studies taking into account age- and sex-matched control groups were of particular interest in this context. We analyzed the factors that influence the levels of said markers in both groups (i.e., the usefulness of the markers), including the age of DS patients, occurrence of regular trisomy 21 or mosaicism, physical activity of patients, and the onset of Alzheimer's disease in DS. This paper was conceived as a handbook—to help for selecting suitable, easy-to-obtain markers for monitoring of the health status of DS patients (e.g., in nutritional studies and during dietary supplementation).

## 1. Introduction

**Figure 1:**
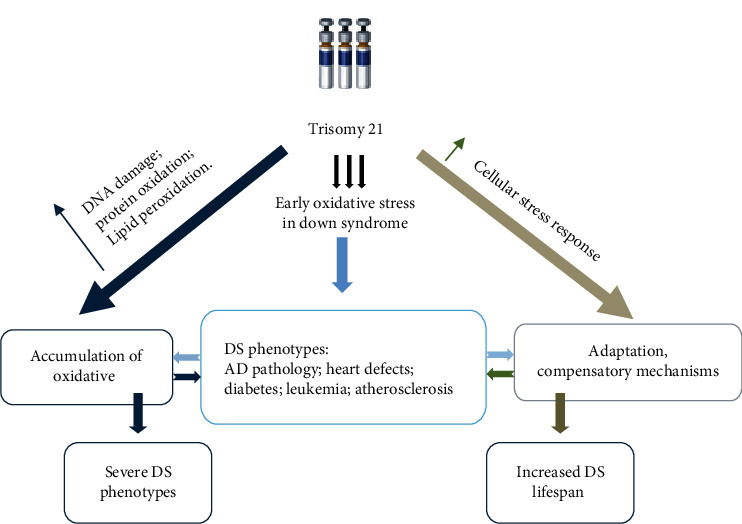
Putative adaptation to oxidative stress in DS. Accumulation of oxidative damage leads to severe phenotypes while the induction of compensatory mechanisms in response to chronic oxidative stress could result in “adaptation” and could contribute to improve the life span of DS subjects.

As discussed by Roizen and Patterson (2003), the overexpression of at least 330 genes, since encoded by the chromosome 21, can play a part in the DS, including 16 genes involved in the mitochondrial energy production, 10 genes with a structural and functional role in the CNS, and 6 genes involved in folate metabolism. The authors underlined some mitochondrial abnormalities in DS patients, which lead to dysfunctions in mitochondrial energy production and metabolism of ROS [1]. Therefore, the cells of a DS patient are permanently subject to inflammation and excessive oxidation incidents which continue throughout their life (Figure 1).

The genetic and physiological mechanisms underlying the origin of ROS overproduction and oxidative stress in DS have been very thoroughly discussed in a number of papers, e.g., in a work of Busciglio and Yankner [2] and the excellent reviews by Barone et al. [3] or Valenti [4].

The presented review, however, does not focus on brain and cerebrospinal fluid oxidation markers as our aim was to identify comparatively easy-to-obtain sources of analytic material. For example, mitochondrial membrane potential (*ΔΨ*) and mitochondrial ultrastructure are affected by excessive oxidation in DS, but the prevalent method of measuring is complex and not really useful in daily clinical practice. Similarly, some sources of samples (fetal brain, brain, amniotic fluid) are difficult to use in human patients on a regular basis. The use of cerebrospinal fluid is not very suitable due to the invasiveness and potential risks of postlumbar puncture headaches. The search for sources of reliable oxidation markers in long-term studies of persons with DS should be focused on sources such as saliva, urine, and blood, where sample collection is relatively simple, noninvasive, and causes little anxiety in patients.

**Figure 2:**
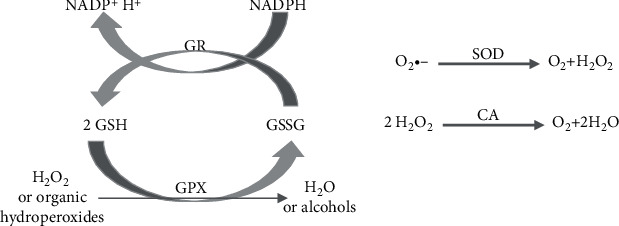
Antioxidant enzymes. CAT: catalase; GPX: glutathione peroxidase; GR: glutathione reductase; H_2_O_2_: hydrogen peroxide; O_2_·^−^: superoxide; SOD: superoxide dismutase.

The system of cellular enzymatic antioxidants (SOD, GPx, CAT, and GR) in DS has been discussed in many papers, e.g., in the excellent review by Pagano and Castello (2012) (Figure 2) [5]. Excessive oxidative stress is caused by elevated levels of Cu/Zn SOD (SOD1) which is coded for on chromosome 21, region 21q22–1, and in DS SOD1 which is overexpressed by approximately 50% relative to control, non-DS patients [6], thus playing an important role in DS-related oxidative stress [7, 8]. As presented in the Supplementary Table 1, most authors reported on the increased GPx activity in DS patients, but some papers evidenced opposite results [9] or similar GPx levels in DS and corresponding matched control groups [10] (Supplementary Table 1).

TAA is a general indicator of the antioxidant status *in vivo*. However, the difference in TAA in DS and non-DS patients can be interesting, instead of values in individual studies. Moreover, comparisons between individual reports are difficult due to the use of various analytical methods applied for the measurement of TAA. Most papers report decreased TAA in samples taken from DS patients, in comparison with non-DS counterparts [11–14] (Table 1). Some studies, however, show that TAA in DS patients was increased, in comparison with healthy controls [15], or no difference was observed [16–18] (Table 1).

Some general information about the status of DS patients may be obtained by monitoring the products of lipid and protein oxidation. TBARS is another possible marker of oxidation. However, conflicting results concerning DS patients were obtained by authors who observed increased [19] as well decreased [20] TBARS levels, whereas in other works, no significant differences in serum lipid peroxides or TBARS were reported [10, 21–23]. MDA can be considered a convenient marker in DS. Authors reported on increased levels of MDA in urine, erythrocytes, plasma, or peripheral blood [9, 24–27] (Supplementary Table 2).

In the past, the level of thiol (sulfhydryl) groups was measured in patients, including those with DS. Glutathione (in various forms) is one of the most important sulfur compounds reflecting the antioxidant status *in vivo*. In numerous works, plasmatic glutathione was decreased in DS patients compared to their healthy counterparts [10, 13, 28, 29]. However, some authors showed insignificant differences in plasma glutathione levels, in comparison with healthy patients [30, 31] (Table 2).

## 2. Factors That Can Affect the Levels of Markers in DS

Some independent factors may influence the outcome of experiments involving a comparison between DS patients and healthy individuals. First of all, genetic factors should be taken under consideration. Cu/Zn SOD, CAT, GPx GR, and MDA levels in DS patients with translocations between chromosomes 14–21, 21–21, and 10–21 were similar to those of age-matched individuals with regular trisomy. The greater the percentage of the normal cell line was present in patients, the lower the oxidative stress observed [32]. De la Torre et al. (1996) reported increased SOD activity in erythrocytes in a population of DS patients with complete trisomy 21 but not in karyotyped persons (by 42% and 28%, respectively). At the same time, the authors observed normal SOD activity in the population with partial trisomy 21, translocations, and mosaicism [33]. MDA levels were dependent on the percentage of diploid and trisomy cells, and Casado, López-Fernández, and Ruiz (2007) observed increased MDA levels in persons with DS as compared to Robertsonian translocation trisomy. Therefore, serum, plasma, or erythrocytic MDA levels seem to be a suitable marker of the oxidative status in DS patients, with the reservation that in the case of DS persons with mosaicism, MDA levels are dependent on the percentage of diploid and trisomy cells (Casado, López-Fernández, and Ruiz 2007) [34].

Physical activity strongly affects the outcome of experiments, as shown in numerous works. It was shown that GPx activity in erythrocytes was significantly elevated after physical training relative to the basal level in DS persons [35, 36]. Erythrocytic (but not serum) SOD activity and erythrocytic and serum CAT activity were increased (in both cases at *p* = 0.05) in the DS group due the physical activity. Moreover, significantly increased TBARs, serum lipid peroxides, and protein carbonyls levels (both at *p* = 0.05) were reported [37]. However, in another study, erythrocytic SOD and GPx activity was significantly decreased after physical training [27]. Increased oxidative stress in the plasma of DS patients after physical training has been reported, including increased MDA and decreased thiol/total proteins ratio in the plasma in the DS group subjects, in comparison with the basal levels registered for the same persons prior the training [38, 39]. The observed levels of plasma carbonyl proteins after the training program in the DS group were increased in comparison with the baseline (the same DS patients before the training, *p* = 0.001). Also, no significant differences in this parameter were reported in healthy controls at the end of the training, compared with baseline (healthy controls before training, *p* > 0.05) [39]. However, certain authors have presented contradictory results. Monteiro et al. (1997) reported on the elevated levels of plasma GSH (*p* = 0.003) in the trained group of patients with DS as compared to the control group of DS subjects not taking part in the training [40]. Zambrano et al. (2009) showed that aerobic exercise caused a significant decrease in the levels of salivary lipid hydroperoxides in the DS group (*p* = 0.001), but had no impact on TAA and nitrite levels [41]. Ordonez and Rosety-Rodriguez (2006) observed reduced plasmatic MDA levels (at the end of the physical training, compared to initial levels) [42]. Aleksander-Szymanowicz et al. (2014) reported on increased GSH levels in venous peripheral blood of adult men with DS after physical training [43].

The age of the patients should also be taken into account when considering the levels of oxidation markers. Campos et al. (2010) observed increased TAA levels in the urine of children with DS (*p* < 0.05) and decreased TAA in the urine of adults with DS (*p* < 0.05), compared to matched, healthy groups. TAA in DS children was increased (*p* < 0.05) as compared to TAA of DS adults [44]. Muchová et al. (2001) reported on significant differences in plasma MDA concentrations in erythrocytes between individuals with DS aged 13–20 years and those over 20 years old (*p* = 0.05) [18]. Licastro et al. (2007) reported decreased levels of plasma peroxides in elderly patients as compared to children and adults with DS (both at *p* < 0.05) [45].

**Figure 3:**
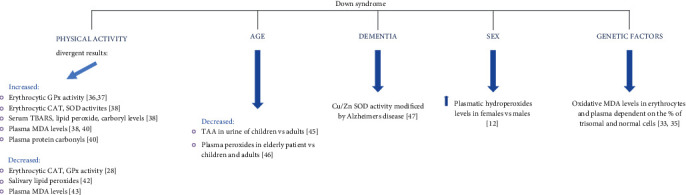
The most significant factors that affect the levels of markers in DS.

In a number of works, dementia or sex was identified as independent factors. Percy et al. (1990) reported on the differences in the increase of erythrocytic Cu/Zn SOD activity in DS patients with and without AD [46]. Massaccesi et al. (2006) showed that plasmatic hydroperoxide levels in females with DS were higher than in the case of males with DS (*p* < 0.05) [12]. The most significant independent factors that affect the levels of markers in DS are summerized in Figure 3.

## 3. Conclusions

In this paper, we provided a considerable number of oxidation/inflammation markers discriminating DS from the diploid state and created a useful tool (handbook) to be used while planning new nutritional experiments. MDA can be considered a convenient marker in DS, as generally agreed by authors. TAA, TBARS, or sulphur levels in DS patients cannot be a reliable marker of oxidation in DS due to the conflicting results found in the original papers cited in this review. Further studies concerning the usability of said selected markers are highly recommendable.

Monitoring of the levels of oxidation markers should account for selected factors interrupting the levels measured in DS patients, mainly the specific type of DS, age, and physical activity of the patient.

To reconcile the conflicting results reported by the authors cited above, a prospective study on a group of DS patients can be proposed in order to measure the marker at the beginning of the supplementation and at the end of the experiment. For example, a precisely planned experiment focused on the effects of antioxidant supplementation on oxidative stress in DS patients should be conducted to examine a larger number of markers. Parisotto et al. (2014) studied the activity of SOD, CAT, GPx, GR, GST, *γ*-glutamyltransferase, and glucose-6-phosphate dehydrogenase, as well as the levels of GSH, UA, TBARS, and protein carbonyls in the peripheral blood of 21 DS patients (3–14 y.o., 7.7 ± 3.18 y., 12 males, and 9 females) and 18 control children (10 males and 8 females; 3–12 y.o., 6.7 ± 3.0 years), before and after daily antioxidant administration over a period of 6 months (vitamin C 500 mg, E 400 mg) [47]. Before the antioxidant therapy, erythrocytic SOD and CAT activity was elevated in DS relative to the control group (by 47% and 24.7%, respectively). Also, GR and *γ*-glutamyltransferase activity was significantly increased (by 98% and 97%, respectively) in DS persons compared to the controls. After supplementation, the erythrocytic GST activity in DS subjects was decreased (61%) compared to the controls. The whole blood concentration of GSH in DS patients was reduced by 27%. The levels of UA in DS persons were higher (by 19%) than in the control. No significant difference between TBARS levels in DS and the control group was observed before supplementation. At the basal state, protein carbonyls were decreased (by 40%) in the DS group relative to the controls. After the 6-month antioxidant supplementation, there was a significant decrease in the GR and *γ*-glutamyltransferase (37%) activity in the supplemented group. The GST erythrocytic activity in DS subjects was significantly increased (44%). After 6 months of antioxidant supplementation, the previously depleted GSH levels (by 27%) were restored. UA levels were unaffected by the antioxidant supplementation but a significant decrease in TBARS (by 181%) was observed in the supplemented DS group. Last but not least, supplementation had no effect on the levels of protein carbonyls in either the DS or the control group [47]. In another work, the same authors monitored biomarkers of inflammation in the peripheral blood of 21 DS patients and 18 children (IL-1*β* and TNF-*α*, TBARS, protein carbonyls, GSH, and UA levels as well as SOD, CAT, GPx, GR, and GST activity) [48]. The authors designed a prospective study involving antioxidant supplementation for 6 months followed by testing, 6 months of interruption, and retesting, followed by antioxidant supplementation for another 6 months and the final retesting.

Following this experimental scheme, the evolution in the levels of oxidation/inflammation markers as well as in the activity of endogenous antioxidants can be continuously monitored in order to verify the effects of the supplementation therapy.

## Figures and Tables

**Table 1:** Markers of the Total Antioxidant Activity reviewed in this work.

Description of groups	The result of the DS group, compared with the corresponding, matched control group of healthy subjects (↑elevated, ↓decreased in DS, in comparison with control)
25 DS persons (18 ± 5 y.o.) and 25 siblings (17 ± 7 y.o.)	↑ Resistance (higher lag time) of serum to lipid oxidation (conjugated dienes formation) (*p* < 0.05) (Nagyová, Sustrová, and Raslová 2000).
40 DS children and 20 apparently healthy control children	↑ Plasma levels of ROS species (*p* < 0.05) ↓ Plasma levels of TAA (*p* < 0.05) (Carratelli et al. 2001).
20 children with DS (10.06 ± 1.04 years) and 18 age-matched control (11.94 ± 0.97 years)	No difference (*p* < 0.05) in plasma TAA (Zitnanová et al. 2006).
23 DS persons (aged 44.1 ± 12.5, 18–58 y.o.), and control subjects (females and males divided into age-matched groups with DS persons): aged 39.6 ± 10.6, 21–60 y.o.) and group of 55 elderly people (aged 66.8 ± 13.4, 61–93 y.o.)	↓ Plasma TAA (Massaccesi et al. 2006).
61 persons with DS (20.76 y.o., 1.67-46.75 y.o.) and 45 age-matched controls (19.57 y.o., 2.67-47.5 y.o.)	No difference in plasma TAA (Muchová et al. 2007).
13 young adults with DS and 15 control patients (both 22 ± 1 y.o.), performing submaximal progressive treadmill exercise (10 min at 30 and 50%, and 20 min at 75% of V O_2max_)	↑ Oxidative stress in plasma of DS persons (+15%; *p* < 0.001) at the end of exercise as well as during recovery after the training but not at rest (*p* = 0.35) (Flore et al. 2008).
32 DS persons (children and adults) and 29 controls; 2 age groups: 19 children with DS (mean age 7.6 ± 3.3 y.o. range 1 - 12 y.o.), 14 healthy age-matched controls (mean age = 9.1 ± 3.0 y.o., range 5 - 13 y.o.), and 13 adults: with DS (mean age = 48.8 ± 4.4 years, 43 -57 y.o.) and 15 healthy age-matched controls (mean age 52.7 ± 5.3 years, 43 - 61 y.o.).	↑ TAA in urine of children with DS (*p* < 0.05). ↓ TAA in urine of adults with DS (*p* < 0.05). Comparison of DS groups: ↑TAA in children than in adults (*p* < 0.05) (Campos et al. 2010).
31 children with DS (3.64 ± 3.39 y.o., 18 boys and 13 girls, divided into 3 groups: less than 4 y.o., 4–8 y.o. and>8 y.o.) with equal number of age and sex-matched controls	↓ Levels of plasma TAA in the whole group of children with DS in comparison with non-DS children (*p* < 0.01). ↓ Levels of plasma TAA in children with DS younger than 4 y.o. (*p* < 0.04) and in children with DS older than 4 y.o. (insignificantly) (Sulthana et al. 2012a).
34 children with DS (7–12 y.o., mean age 9.44 y.o., 19/15 males/females) and 34 control, age-matched children (7–12 y.o., mean age 9.29 y.o., 13/21 males/females)	↓ TAA activity of saliva (especially boys with DS in comparison with control boys, *p* < 0.001) (Subramaniam et al. 2014).
30 patients with DS (14–24 y.o.) and 30 age-matched control subjects	No statistical difference (*p* < 0.05) in the TAA of saliva in both groups (de Sousa et al. 2015).

**Table 2:** Sulfur compounds reviewed in this work.

Description of groups	The result of the DS group, compared with the corresponding, matched control group of healthy subjects (↑elevated, ↓decreased in DS, in comparison with control)
8 young male adults with DS, performing physical training (10 min warm-up, aerobic session at a work intensity of 60–75% of VO_2_ peak lasting from 15 to 25 min, increasing 5 min every 5 weeks and by a 5 min cool-down period, 3 days/week), 8 young male adults with DS in the control group, compared with the healthy population	↑ Plasma GSH levels of the trained group compared with the control group with DS (*p* = 0.003) (Monteiro et al. 1997).
42 children with full (caryotypically confirmed) trisomy 21 and 36 non-DS siblings (mean age 7.4 ± 4.2 y.o.)	↓ Plasmatic Hcy, methionine, S-adenosylhomocysteine and S-adenosylmethionine ↓ Plasmatic glutathione (*p* < 0.01) (Pogribna et al. 2001).
A male child with trisomy 21	↓ Plasmatic Hcy in comparison with healthy population (Al-Gazali et al. 2001).
40 DS children and 20 apparently healthy control children	↓ Levels of thiols (sulphydryl groups) (*p* < 0.05) (Carratelli et al. 2001).
Studied 60 children with DS (3.6 ± 3.33 years; range 0.5–12 years, 43% females and 57% males) and 29 siblings without DS (7.3 ± 4.48 years; range 1–17 years, 51% females and 48% males)	Insignificant (*p* > 0.05) differences in plasma GSH as well as GSSG levels (Pinto et al. 2002).
12 patients with DS and 12 age and sex-matched persons in the control group	No significant differences in the levels of GSH in sera (Cengiz, Seven, and Suyugűl 2002).
46 children with DS (26 females and 20 males; 6.7 ± 2.7 y.o.) and 64 patients without DS (randomly selected 30 males, 34 females; 5.1 ± 2.3 y.o.)	↓ Of all glutathione forms in blood: glutathionyl-haemoglobin (by 44%), GSH + GSSG (by 30%), and GSH (by 25%) (Pastore et al. 2003).
44 persons with DS (mean age 23.2 y.o.) in comparison with 26 control patients (mean age 23.3 y.o.).	↓ GSSG concentration (*p* = 0.012) ↓ GSH concentration (*p* = 0.064) No difference in the ratio of GSH/GSSG (*p* = 0.848) (Garaiová et al. 2004).
32 DS patients, 18 females and 14 males, 2 months-57 years (median age = 21 yrs.; 22.3 ± 18.2 y.o.), and 67 control subjects in the same age range (median age = 16 years; 21.4 ± 14.4 y.o.)	↓ Plasma GSH levels in the group < 15 years (not significantly) ↑ Plasma GSH levels in the group > 15 years (*p* = 0.05) ↑ Plasma GSSG levels in the group < 15 years (*p* = 0.006) ↓ Plasma GSSG levels in the group > 15 years (not significantly) ↑ GSSG:GSH ratio in young patients with DS (<15 years) ↓ GSSG:GSH ratio in patients with DS aged > 15 years (Pallardó et al. 2006).
13 DS patients (male, average age 60 years) and 20 age-matched individuals	↑ Plasmatic tHcy levels (*p* < 0.05) (Licastro et al. 2006).
61 persons with DS (20.76 y.o., 1.67-46.75 y.o.) and 45 age-matched controls (19.57 y.o., 2.67-47.5 y.o.)	↓ Of plasma GSH (*p* = 0.018) No difference in plasma GSSG levels (Muchová et al. 2007).
13 young adults with DS and 15 healthy control patients (both 22 ± 1 y.o.), performing submaximal progressive treadmill exercise (10 min at 30 and 50%, and 20 min at 75% of V O_2max_)	↓ Thiols/total proteins ratio in plasma during trainings and recovery (*p* < 0.001) (Flore et al. 2008).
31 children with DS (3.64 ± 3.39 y.o., 18 boys and 13 girls, divided into 3 groups: less than 4 y.o., 4 – 8 y.o. and>8 y.o.) with equal number of age and sex-matched controls	↓ Erythrocytic GSH levels in children younger than 8 y.o. (not significantly) and in children older than 8 y.o. (at *p* < 0.002) (Sulthana et al. 2012a).
35 persons with DS (median 10–90^th^ percentile) aged 11.0 y.o. (1.9–27.0 y.o., 20 males) and control group of 47 healthy children and adolescents (median (10–90^th^ percentile) aged 13.0 y.o. (5.7–17.0 y.o., 21 males)	↑ Plasma levels of *S*-adenosylhomocysteine and *S*-adenosylmethionine (by 51% and 34%, respectively, both at *p* < 0.001) ↓ Total serum Hcy (by 29%) (*p* < 0.001) (Obeid et al. 2012).
20 DS persons (10 males and 8 females; 3–12 years, mean age 7.7 ± 3.18 y.o.) and 18 control subjects (6.7 ± 3.0 y.o.)	↓ Serum GSH levels (24.9%) (Garlet et al. 2013).
Nontrained 15 men with DS (21–24 y.o., mean age 22.4 ± 0.9 y.o.) taking part in a six-week aerobic training (3 times a week for 6 weeks, 10 min warm-up, 20–25 min of the main phase at work intensity of 60–75% of max. Peak heart rate calculated as 194.5 (0.56×age, 10-minute cool down)	↑ levels of GSH in venous peripheral blood (*p* = 0.00099) (Aleksander-Szymanowicz et al. 2014).

## Data Availability

Not applicable-it is a review.

## Abbreviations

ABTS: 2,2′-azino diethylbenzothiazo-line sulfonic acid
AD: Alzheimer's disease
CAT: Catalase
CNS: Central nervous system
CRP: C-reactive protein
DS: Down syndrome
GPx: Glutathione peroxidase
GR: Glutathione reductase
GSH: Reduced glutathione
GS-Hb: Glutathionyl-hemoglobin
GSSG: Oxidized glutathione
GST: Glutathione S-transferase
Hb: Hemoglobin
Hcy: Homocysteine
oxLDL: Oxidized low density lipoprotein
MDA: Malondialdehyde
ROS: Reactive oxygen species
SOD: Superoxide dismutase
TAA: Total antioxidant activity
TBARS: Thiobarbituric acid reactive substances
tHcy: Total homocysteine
UA: Uric acid
VLDL: Very low-density lipoproteins.

## Supplementary Materials

Supplementary Materials Supplementary Table 1: endogenous enzymes reviewed in this work. Supplementary Table 2: lipid and protein oxidation products reviewed in this work.
