# Supplementary material for: Peripheral Oxidation Markers in Down Syndrome Patients: The Better and the Worse
Source: Dis Markers. 2021 Jun 28;2021:5581139. doi: 10.1155/2021/5581139 (PMC8260317; doi:10.1155/2021/5581139)
Supplement: Supplementary Materials — Supplementary Table 1: endogenous enzymes reviewed in this work. Supplementary Table 2: lipid and protein oxidation products reviewed in this work. [file 5581139.f1.zip › Table 1 Supplementary.docx]

Supplementary Table 1: Endogenous enzymes reviewed in this work.

| Description of groups | The result of the DS group, compared with the corresponding, matched control group of healthy subjects (↑ elevated, ↓ decreased in DS, in comparison with control): |
| --- | --- |
| 29 persons with DS and 32 age-matched controls | ↑ Plasma SOD1 and GPx levels in the erythrocytes (both at *p* < 0.001) [49]. |
| 14 persons with DS (5 females/9 males) and age-matched healthy donors (23 females/58 males). | ↑ Erythrocytic activity of SOD and GPx (both at *p* < 0.001)  no differences in the CAT activity [50]. |
| 34 adult DS patients (31–70 yrs.), and healthy control individuals matched for sex, age (± 2 yrs.) | ↑ Erythrocytic GPx activity (1.25-fold)  ↑ erythrocytic Cu/Zn SOD activity in DS patients without AD (1.39 –fold) and in DS patients with AD (1.16 –fold)  no difference in CAT activity [46]. |
| 260 DS patients (123 females/137 males), 2–45 y.o., 57 patients with complete trisomy 21, 51 patients with partial trisomy 21, translocations and mosaicisms, and 152 not karyotyped) | ↑ SOD activity in erythrocytes in the DS population with complete trisomy 21 and not karyotyped (by 42% and 28%, respectively)  normal SOD activity in the population with partial trisomy 21, translocations and mosaicism [33]. |
| 8 young male adults with DS, performing physical training (10 min warm-up, aerobic session at a work intensity of 60 – 75% of VO2 peak lasting from 15 – 25 min, increasing 5 min every 5 weeks and by a 5 min cool-down period, 3 days/week), 8 young male adults with DS not performing the task, compared with healthy population | ↑ SOD activity (by 1.4x) [40]. |
| 72 patients with DS (mean age 17.8 ± 15.8 y.o.) and 72 persons in the control group without DS (mean age 14.6 ± 10.8 y.o.) | ↑ Erythrocytic SOD1, CAT, GPx, and GR activity (significantly) [51]. |
| 37 persons with DS (1–20 yrs.) as compared to 35 control subjects without DS (siblings of children with DS) both groups divided into 1–<6 years, 6–<13, 13–<20 and over 20 years. | ↑ Erythrocytic activity of Cu/Zn SOD and GPx (*p* < 0.05)  ↑ SOD/CAT + GPx ratio  no significant differences in CAT and GR activity [18]. |
| 12 patients with DS and 12 age and sex-matched persons in the control group | ↑ Serum levels of SOD and GPx (significantly)  no significant changes in serum GST [31]. |
| 46 children with DS (26 females and 20 males; 6.7 ± 2.7 y.o.) and 64 patients without DS (randomly selected 30 males, 34 females; 5.1 ± 2.3 y.o.) | ↑ Erythrocytic levels of SOD/GPx activity ratio (by 53%)  ↑ erythrocytic levels of SOD activity (by 34%)  no difference in GR activity [29]. |
| 44 persons with DS (mean age 23.2 y.o.) in comparison with 26 control patients (mean age 23.3 y.o.). | ↑ Erythrocytic activity of SOD and GPx  ↑ the ratio of SOD/(GPx + CAT) (*p* = 0.006)  no significant difference in the CAT activity  [25]. |
| 25 female, 25 male persons with DS (3-24 yrs., 14.20 ± 6.62 y.o.) with total chromosome 21 trisomy | ↑ Serum SOD and CAT activity (at *p* = 0.004 and 0.002, respectively) [19]. |
| 100 individuals with DS: newborns to 29 y.o., 34 males and 66 females and a group of age-matched persons without DS: 40 males, 60 females | ↑ Levels of SOD activity in the whole blood (*p* < 0.05)  ↑ activity of erythrocytic GPx (*p* < 0.05)  no significant difference in the erythrocytic GR and CAT activity in hemolysates [32]. |
| 31 male adolescents with DS (mean age 16.3 ± 1.1 y.o.) and the control group (17 healthy siblings of persons with DS, mean age 16.6 ± 1.3 y.o.) | ↑ Erythrocytic levels of SOD, GPx, and glucose-6-phosphate dehydrogenase (by 35.2%, 15.3%, and 14.9%, respectively) (at *p* = 0.019, 0.03, and 0.038, respectively)  unchanged CAT activity (*p* = 0.151) [52]. |
| 31 adolescents with DS (16.3 ± 1.1 y.o.) taking part in a 12-week training program (3 days/week, including warm-up, exercise at 60–75% intensity of the peak heart rate calculated from 194.5 – (0.56 × age), and a cool-down period) | ↑ Erythrocytic SOD activity (*p*  = 0.099)  ↑erythrocyte GPx pre-exercise and post-exercise enzyme activity in comparison with a basal level (*p* = 0.011) [53]. |
| 61 persons with DS (20.76 y.o., 1.67-46.75 y.o.) and 45 age-matched controls (19.57 y.o., 2.67-47.5 y.o.) | ↑ Of blood SOD activity by 149.6% [54]. |
| 21 male individuals with DS (23.3 ± 2.1 y.o, body mass index of 23.0 ± 1.2 kg/m^2^) taking part in a 16-week supervised exercise program (3 weekly 50 min. aerobic sessions of adapted judo training, prescribed and monitored on lactate threshold. | ↑ Erythrocytic but not serum SOD activity (*p* = 0.05)  ↑ erythrocytic and serum CAT activity (*p* = 0.05)  serum GPx activity was unaltered [37]. |
| 15 patients with DS without congenital heart disease (CHD) (8.4 ± 1.0 y.o.), 11 patients with DS with CHD (6.7 ± 1.5 y.o.), 10 patients with CHD without DS (8.2 ±1.5 y.o.), and 16 control patients (7.8 ± 1.0) | ↑ Activity of SOD and GPx in neutrophils of all patients with DS (*p* < 0.05 and 0.01, respectively)  no significant difference in CAT activity [55]. |
| 20 male adolescents with DS (14.86 ± 7.07 y.o., height 129.00 cm ± 7.28, 46.39 kg ± 16.79, BMI 31.92 ± 6.83, WC 87.12 ± 3.5, WHR (N< 1) 0.87 ± 0.15) who took part in a 12-week training program. | ↓ Erythrocytic SOD (*p* = 0.019) and GPx (*p* = 0.05) activity  no difference in the erythrocytic CAT activity (*p* = 0.373) [27]. |
| 30 adolescent males with DS (15–18 y.o.) performing electronic treadmill exercise (12 weeks) and 30 healthy subjects. | ↓ Serum GPx activity (*p ≤* 0.001)  **In DS group:**  ↑ serum GPx activity after the training (*p* ≤ 0.001) [36]. |
| 20 DS persons (10 males and 8 females; 3–12 years, mean age 7.7 ± 3.18 y.o.), and 18 control subjects (6.7 ± 3.0 y.o.) | ↑ Activity of serum SOD (47.2%), CAT (24.7%) and GR (49.6%)  ↓ serum GST activity (61.2%)  no significant difference in serum GPx activity [10]. |
| 28 persons with DS (12 women, 26.2 ± 5.76 y.o. /16 men, 28.8 ± 6.94 y.o.) and 28 matched by age and sex control subjects (15 women, 24.12 ± 5.54 y.o./13 men, 23.67 ± 3.98 y.o.) | ↑ SOD and CAT activity in the whole blood (by 42% and  31% respectively, at both *p* = 0.033) [20]. |
| 30 patients with DS (14–24 y.o.) and 30 age-matched control subjects in control group | ↑ Salivary SOD activity (*p* < 0.05) [17]. |
| A cross-sectional study of age-, gender- and peripheral blood profile-matched 36 children with DS (mean age 1 y.o., males/9 females) and 40 controls without DS (mean age 2.2 y.o., 28 males/12 females) | ↑ Total SOD activity in peripheral blood (*p* ≤ 0.001)  ↓ extracellular GPx activity in peripheral blood (*p* = 0.033) [9]. |
