# Supplementary material for: Peripheral Oxidation Markers in Down Syndrome Patients: The Better and the Worse
Source: Dis Markers. 2021 Jun 28;2021:5581139. doi: 10.1155/2021/5581139 (PMC8260317; doi:10.1155/2021/5581139)
Supplement: Supplementary Materials — Supplementary Table 1: endogenous enzymes reviewed in this work. Supplementary Table 2: lipid and protein oxidation products reviewed in this work. [file 5581139.f1.zip › Table 2 Supplementary.docx]

Supplementary Table 2: Lipid and protein oxidation products reviewed in this work.

| Description of groups | The result of the DS group, compared with the corresponding, matched control group of healthy subjects (↑elevated, ↓decreased in DS, in comparison with control): |
| --- | --- |
| 8 young male adults with DS, performing physical training (10 min warm-up, aerobic session at a work intensity of 60 – 75% of VO_2_ peak lasting from 15 – 25 min, increasing 5 min every 5 weeks and by a 5 min cool-down period, 3 days/week), 8 young male adults with DS in the control group, compared with healthy population | ↑ Plasma TBARS in the trained group in comparison with non-trained DS group (*p* = 0.034) [40]. |
| 85 DS patients (5.11 ± 4.15 y.o.) and 81 control siblings (7.56 ± 4.67 y.o.) | ↑ Urinary levels of MDA (*p* = 0.0033) [24]. |
| 37 persons with DS (aged 1.7 to 46.8) as compared to 35 control subjects without DS (siblings of children with DS), both groups divided into 1–<6 years, 6–<13, 13–<20 and over 20 years. | ↑ Serum levels of MDA (*p* = 0.021)  significant differences in plasma MDA concentration in DS persons aged 13–<20 years and over 20 years (*p* = 0.05) [18]. |
| 3 groups of children with DS (1–8 y.o.): 46 persons with trisomy 21 or complete translocation, 30 children with cognitive impairment and without DS, and 43 developmentally healthy control subjects | ↑ Levels of intramitochondrial superoxide (*p* < 0.03),  no significant differences in serum lipid peroxides or TBARS [21]. |
| 44 persons with DS (mean age 23.2 y.o.) in comparison with 26 control patients (mean age 23.3 y.o.). | ↑ Levels of erythrocytic MDA (*p* = 0.019) [25]. |
| 100 individuals with DS: newborns to 29 y.o., 34 males and 66 females and a group of age-matched persons without DS: 40 males, 60 females | ↑ Levels of MDA (*p* < 0.02)  the greater the percentage of the normal cell line was present in patients, the lower oxidative stress was observed [32]. |
| 25 female, 25 male persons with DS (3-24 yrs., 14.20 ± 6.62 y.o.) with total chromosome 21 trisomy | ↑ Plasma levels of TBARS (*p* < 0.02) [19]**.** |
| 31 male adolescents with DS who took part in a 12-week physical training program (3 days/week at a work intensity of 60–75% of max. peak heart rate HR_max_=194.5), no control group of healthy patients | ↓ Plasmatic levels of MDA (at the end of the experiment, in comparison with the initial levels) [35]. |
| 23 DS persons (aged 44.1 ± 12.5, 18–58 y.o.), and control subjects (females and males divided into age-matched groups with DS persons): aged 39.6 ± 10.6, 21–60 y.o.) and group of 55 elderly people (aged 66.8 ± 13.4, 61–93 y.o.) | ↑ Plasma levels of hydroperoxides in DS group compared with age-matched control (*p* < 0.01)  **Comparison of DS subjects:**  ↑ hydroperoxide levels in females than males (p < 0.05) [12]. |
| DS patients: 23 children (age 2–14 years); group 2 of 14 adults (age 20–50 years) and group 3 of 13 elderly patients (>60 years), and a group of 20 age-matched healthy  controls. | No differences in the plasmatic advanced protein oxidation products (*p* > 0.05) [56]. |
| 20 children with DS (10.06 ± 1.04 years) and 18 age-matched (11.94 ± 0.97 years) | ↑ Plasma levels of protein carbonyls (*p* < 0.01)  no difference in plasma hydroxyl-2-nonenal [16]. |
| 100 persons with DS (34 males and 66 females, newborn to 29 years, divided into 6 age groups, including 90 individuals with regular trisomy 21, four persons with trisomy 21 by Robertsonian translocation and 6 persons with mosaic trisomy 21) and the control group (age-matched 40 males and 60 females persons). | ↑ Erythrocytic MDA levels in all age groups (*p* < 0.05)  ↑ erythrocytic MDA levels in persons with DS compared with Robertsonian translocation trisomy [34]. |
| 61 persons with DS (20.76 y.o., 1.67-46.75 y.o.), 45 age-matched controls (19.57 y.o., 2.67-47.5 y.o.) | No significant difference in plasmatic MDA levels [54]. |
| 23 children with DS (2–14 y.o.), 14 adults with DS (20–50 y.o.), 13 elderly patients with DS (>60 years) and a group of 20 controls (5–18 years) | ↑ Plasma levels of oxLDL in children and adults with DS (20–50 years) (*p* < 0.05 and *p* < 0.01, respectively)  ↑ plasma levels of oxLDL in elderly patients with DS (*p* > 0.05)  ↑ plasma levels of peroxides (*p* < 0.05) in children and adults with DS  **Comparison of DS patients:**  ↓ plasma levels of peroxides in elderly patients than in children as well as in adults with DS (20–50 years) (both at *p* < 0.05) [45]. |
| 13 young adults with DS and 15 control patients, performing submaximal progressive treadmill exercise (10 min at 30 and 50%, and 20 min at 75% of V O_2max_) | ↑ Plasma levels of MDA (*p* < 0.01) [38]. |
| 21 male individuals with DS (23.3 ± 2.1 y.o, body mass index of 23.0 ± 1.2 kg/m^2^) taking part in a 16-week supervised exercise program (3 weekly 50 min. aerobic sessions of adapted judo training, prescribed and monitored on lactate threshold. | ↑ TBARS in erythrocytes and serum lipid peroxides in adults with DS after judo training (*p* < 0.05)  ↑ erythrocytic and serum protein carbonyls levels in blood (*p* = 0.05) [37]. |
| 7 males and 5 females with DS (18.42 ± 1.89 y.o.) who performed a 1,600-m walking test | ↓ Lipid hydroxyperoxides in saliva immediately after the walking (*p* < 0.001)  but TAA was not influenced by the exercise [41]. |
| 26 children with DS (7 of them taking levothyroxine for hypothyroidism, 13 males and 13 females, 9.0± 3.7 (3–14 y.o.) and their 19 non-DS siblings (8 males, 11 females, 9.6±3.3 (5–14 y.o.). Two age groups: <10 years and ≥10 years). | No difference between urinary levels of TBARS [22]. |
| 78 adolescents and adults with DS (54 patients without hyperthyroidism and 24 taking levothyroxine for hyperthyroidism), and 65 controls | No difference between urinary levels of TBARS [23]. |
| 31 children with DS (3.64 ± 3.39 y.o., 18 boys and 13 girls, divided into 3 groups: less than 4 y.o., 4–8 y.o. and 8–14 y.o.) with equal number of age and sex-matched controls | ↑ Of plasmatic protein carbonyls in the whole group of DS children and in any age groups 4–8 y.o. and 8–14 y.o. (at *p* < 0.001, *p* = 0.0065 *p* = 0.0022, *p* < 0.001, respectively)  ↑ of plasmatic MDA in the whole group of DS children and in age groups 4–8 y.o. and 8–14 y.o. (at *p* < 0.001, *p* < 0.002, *p* = 0.0102, respectively) [26]. |
| 20 male adolescents with DS (14.86 ± 7.07 y.o., height 129.00 cm ± 7.28, 46.39 kg ± 16.79, BMI 31.92 ± 6.83, WC 87.12 ± 3.5, WHR (N< 1) 0.87 ± 0.15) who took part in a 12-week training program. | ↑ Erythrocytic levels of MDA (*p* = 0.02) [27]. |
| 31 adolescents with DS taking part in a 12-week training program (on a treadmill, 3 sessions/week, consisting of 15 min warm-up, a main part 20–35 min (increasing 5 min each 3 weeks), at a work intensity of 60–75% of peak heart rate), and a 7 age-, sex- and BMI-matched DS patients (control not subjected to any training program) | ↑ Plasma carbonyl proteins after the training program in DS group, compared with baseline (*p* = 0.001)  no significant differences in carbonyl proteins in controls at the end of training compared with baseline (*p* > 0.05) [39]. |
| 30 adolescent males with DS, 15–18 y.o.) performing electronic treadmill exercise (12 weeks) and 30 healthy subjects. | ↑ Serum levels of MDA (*p* ≤ 0.001)  **In DS group:**  ↓ Serum levels of MDA after the training (*p* = 0.01) [36]. |
| 20 DS persons (10 males and 8 females; 3–12 years, mean age 7.7 ± 3.18 y.o.), and 18 control subjects (6.7 ± 3.0 y.o.) | No significant differences in serum levels of TBARS  ↓ levels of protein carbonyls (by 31.7%) [10]. |
| 28 persons with DS (12 women, 26.2 ± 5.76 y.o. /16 men, 28.8 ± 6.94 y.o.) and 28 matched by age and sex control subjects (15 women, 24.12 ± 5.54 y.o./13 men, 23.67 ± 3.98 y.o.) | ↓ Of plasma TBARS and total thiol content by 47% and 16% respectively (*p* < 0.05) [20]. |
| 30 patients with DS (14–24 y.o.) and 30 age-matched control subjects in control group | ↑ Salivary levels of MDA (*p* < 0.05) [17]. |
| A cross-sectional study of age-, gender- and peripheral blood profile-matched 36 children with DS (mean age 1 y.o., males/9 females) and 40 controls without DS (mean age 2.2 y.o., 28 males/12 females) | ↑ MDA levels in peripheral blood (*p* = 0.013) [9]. |
